# Identification of Novel SNP in Promoter Sequence of *TaGW2-6A* Associated with Grain Weight and Other Agronomic Traits in Wheat (*Triticum aestivum* L.)

**DOI:** 10.1371/journal.pone.0129400

**Published:** 2015-06-15

**Authors:** Vandana Jaiswal, Vijay Gahlaut, Saloni Mathur, Priyanka Agarwal, Manoj Kumar Khandelwal, Jitendra Paul Khurana, Akhilesh Kumar Tyagi, Harindra Singh Balyan, Pushpendra Kumar Gupta

**Affiliations:** 1 Department of Genetics and Plant Breeding, Ch. Charan Singh University, Meerut, India; 2 Interdisciplinary Centre for Plant Genomics, University of Delhi South Campus, New Delhi, India; 3 Indian Agricultural Statistics Research Institute, Pusa Campus, New Delhi, India; 4 Department of Plant Molecular Biology, University of Delhi South Campus, New Delhi, India; 5 National Institute of Plant Genome Research, New Delhi, India; National Institute of Plant Genome Research (NIPGR), INDIA

## Abstract

*TaGW2* is an orthologue of rice gene *OsGW2*, which encodes E3 RING ubiquitin ligase and controls the grain size in rice. In wheat, three copies of *TaGW2* have been identified and mapped on wheat homoeologous group 6 viz. *TaGW2-6A*, *TaGW2-6B* and *TaGW2-6D*. In the present study, using as many as 207 Indian wheat genotypes, we identified four SNPs including two novel SNPs (SNP-988 and SNP-494) in the promoter sequence of *TaGW2-6A*. All the four SNPs were G/A or A/G substitutions (transitions). Out of the four SNPs, SNP-494 was causal, since it was found associated with grain weight. The mean TGW (41.1 g) of genotypes with the allele SNP-494_A was significantly higher than mean TGW (38.6 g) of genotypes with the allele SNP-494_G. SNP-494 also regulates the expression of *TaGW2-6A *so that the wheat genotypes with SNP-494_G have higher expression and lower TGW and the genotypes with SNP-494_A have lower expression but higher TGW. Besides, SNP-494 was also found associated with grain length-width ratio, awn length, spike length, grain protein content, peduncle length and plant height. This suggested that gene *TaGW2-6A* not only controls grain size, but also controls other agronomic traits. In the promoter region, SNP-494 was present in ‘C**G**CG’ motif that plays an important role in Ca^2+^/calmodulin mediated regulation of genes. A user-friendly CAPS marker was also developed to identify the desirable allele of causal SNP (SNP-494) for use in marker-assisted selection for improvement of grain weight in wheat. Using four SNPs, five haplotypes were identified; of these, Hap_5 (G_A_G_A) was found to be a desirable haplotype having significantly higher grain weight (41.13g) relative to other four haplotypes (36.33-39.16 g).

## Introduction

Improvement in average grain yield in wheat has attracted the attention of wheat breeders all over the world, and several initiatives in this direction have recently been taken, both at the national and international levels. These include Wheat Inititaive (www.wheatinitiative.org), Wheat Yield Network (WYN; www.bbsrc.ac.uk/web/FILES/Resources/wheat-yield-network.pdf) and its International Wheat Yield Partnership (IWYP; iwyp.org) program and 20:20 program of the UK (‎www.rothamsted.ac.uk/ our-science/2020-wheat). One of the most important traits contributing to grain yield is grain size (thousand grain weight = TGW), which is also related to higher flour recovery [1–2] and milling quality of grain. Keeping this in view, grain size in wheat has been one of the targets for selection both during domestication and modern wheat breeding [3–4].

Grain weight is a polygenic trait and is controlled by a large number of genes/QTL that are distributed on all wheat chromosomes [2, 5–21]. Among these genes, three genes that are orthologous to rice gene *OsGW2* were earlier identified and mapped on three chromosomes of the homoeologous group 6; these were described as *TaGW2-6A*, *TaGW2-6B*, *TaGW2-6D* [22]. Two SNPs (-593A/G and -739G/A) were also earlier reported in the promoter region of the gene *TaGW2-6A*. One of the two SNPs (-593A/G) in the promoter region of *TaGW2-6A*, and an insertion of a single T-base in the eighth exon of this gene (detected in a large kernel wheat variety Lankaodali) were shown to be associated with grain size [22–23]. Although, a negative correlation between expression of *TaGW2* and grain size was observed in two earlier studies [22–23], a positive correlation was suggested in another study, where knocking out of the gene using RNA interference (RNAi) involving reduction in *TaGW2* transcript levels, led to reduction in endosperm cell number associated with reduction in grain size [24]. Further studies may be needed to resolve this apparent contradiction in the results. Biochemical and molecular analyses revealed that *TaGW2-6A* encodes a functional E3 RING ubiquitin ligase with nucleocytoplasmic subcellular partitioning.

In the present study, we analysed sequence polymorphism in the promoter region of *TaGW2-6A* in a collection of 207 Indian wheat genotypes. Interestingly, we found two novel SNPs (one SNP present in CGCG motif) in the promoter region along with two other SNPs that were also reported by Su et al. [22]. A study of association of these SNPs and that of the corresponding haplotypes with TGW in Indian wheat genotypes allowed identification of a novel causal SNP and a causal haplotype. The causal SNP also modulated the expression of the gene *TaGW2* in developing grains so that the negative regulation of the gene expression was associated with higher grain weight. A functional marker (cleaved amplified polymorphic sequence—CAPS) was also developed for identification of individual alleles of causal SNP for use in wheat breeding programs aimed at grain weight improvement.

## Materials and Methods

### Plant material and recording of data on grain size and other agronomic traits

The plant material used in this study comprised as many as 207 Indian wheat genotypes, released during 1910–2006 for commercial cultivation in different agro-climatic regions of India. The seed of the above genotypes was procured from the Indian Institute of Wheat and Barley Research (IIWBR), Karnal (India). The data on TGW, grain width, grain length, length-width ratio, and five other agronomic traits recorded on the above 207 Indian wheat genotypes were used in the present study.

Each metric observation was based on an average of 10 randomly selected plants. The observations and data on different traits were recorded in the following manner: (i) 1000-grain weight (TGW); weight of 1000 grains expressed in grams; (ii) grain-length; recorded in milimeter using software SmartGrain, (iii) grain-width; recorded in milimeter using software SmartGrain (iv) grain length-width ratio; recorded using software SmartGrain; (v) awn length; measured in cm from middle one-third region of the ear; (vi) spike length; measured in cm from the base of the ear to the tip of the apical spikelet (excluding awns); (vii) grain protein content: estimated using Food and Feed Analyzer NIR 1255; (viii) peduncle length: measured in cm from base (collar) of the spike to the first node; and (ix) plant height: measured in centimetre (cm) from base of the plant to the tip of the spike (excluding awns) of the longest tiller. Data for grain length, width and length-width ratio were recorded during present study, and those for the remaining traits were procured from IIWBR, Karnal [25]; the data at IIWBR was generated in evaluation trials, conducted for DUS traits, during three consecutive years (2003–04 to 2005–06) at Karnal, India.

### DNA isolation and PCR amplification

For each genotype, genomic DNA was extracted from the leaves of one month-old plants using a modified CTAB method [26]. Isolated DNA was purified by RNase A treatment and phenol: chloroform: isoamyl alcohol precipitation following Sambrook et al. [27]. The quality and quantity of DNA were checked on agarose gel through a comparison with known quantities of λ Hind III DNA marker. The gene-primers that were specific for the sub-genome A (Hap-6A-P1_For and Hap-6A-P1_Rev) reported earlier were used to amplify the promoter region of gene *TaGW2-6A* [22]. PCR reactions were performed using a total volumes of 15 μl, with 3 pmol of each primer, 120 μM of each dNTP, 80 ng genomic DNA, 0.75 unit Jumpstart Accu Taq La DNA polymerase and 2 μl 10× buffer (Catalog number B0174), Sigma, USA. The PCR was carried out using Veriti Thermal Cycler, Applied Biosystem using the following profile with a ramp rate of 3.35°C/second: initial denaturation at 95°C for 3 min, followed by 32 cycles at 95°C for 30s, annealing at 58°C for 30s, and extension at 72°C 30s, with a final extension at 72°C for 10 min. PCR products were resolved by electrophoresis on 2% agarose gels.

### Sequencing of PCR product

For sequencing of PCR products, approximately 500 ng of each PCR products obtained above were used and cleaned using the following reaction. 1 U Shrimp Alkaline Phospatase (Fermentas) and 10 U of Exonuclease I (Fermentas) in a final volume of 10 μl at 37°C for 15 min followed by enzyme inactivation at 85°C for 15 min.

One μl (~50 ng) of each of the above cleaned samples was directly used as template for sequencing. The reaction was set-up using 10 pmole primer and 0.5 μl Big-dye chemistry v3.1 (ABI) in a final volume of 10 μl. The sequence of cycles was set-up with the following profile at a ramp rate of 3.35°C/second: denaturation at 96°C for 10s, primer annealing at 50°C for 5 s and extension at 60°C for 4 min for a total of 30 cycles. Gene Amp PCR system 9700 (Applied Biosystem) was used for PCR amplification. The fluorescently labelled PCR products were analysed using an ABI 3730xl sequencer.

### Sequence alignment and SNP detection

Sequence alignment and SNP detection were performed using software CLC genomics/DNA workbench (http://www.clcbio.com). In order to identify quality SNPs, specific criteria based on the read depth, minor allele frequency and the quality of flanking regions were used. Each high quality SNP was identified in a segment of appropriate size, where all bases matched except the SNP identified, so that a 15-bp flanking region on each side of an identified SNP had no extra SNPs or indels [28, 29]. Only SNPs with minor allele frequency of no less than 5% in the population were declared as quality SNPs.

### Marker-trait association

Descriptive statistics for all nine traits including TGW were obtained using SPSS. Association analysis was conducted using General Linear Model (GLM) with 1000 permutations with the help of software TASSEL (http://www.maizegenetics.net). Significance of the association was determined by p-value (<0.05). Mann-Witney (non-parametric test) was applied to test the significance of difference for TGW between the two allele classes of each SNP locus using SPSS. Analysis of variance (ANOVA) was conducted by PROC GLM in the Statistical Analysis System (SAS Institute, 1997) to test the significant differences of TGW among different haplotypes.

### RNA extraction and qRT PCR

Total RNA was extracted from immature seed (15 DAP = days after pollination) from 10 genotypes (5 genotypes with SNP-494_A and 5 genotypes with SNP-494_G) using Sigma Aldrich’s Spectrum Plant Total RNA kit. Quantitative Real-time PCR (qRT-PCR) was used to analyze the transcript level of *TaGW2-6A* (primer sequences: *TaGW2-6A*_For: AAGCATGGGTGCTGCGGAA, *TaGW2-6A*_Rev: GTCAGCAAAAGGCAACGGTA [30]). qRT-PCR was performed with Thermo Scientific’s DyNAmo Flash SYBR Green qPCR kit, using Applied Biosystem’s 7500 Fast RT-PCR System according to the manufacturer’s instructions. qRT-PCR reaction was set up with the following thermal profile using a ramp at the rate of 3.5°C/second: 95°C for 15 min (initial denaturation), followed by 40 cycles with 95°C for 10 s (denaturation) and 60°C for 30 s (annealing/extension). The relative transcript level of *TaGW2-6A* was calculated using 2^− ΔΔCT^ method [31]. *TaActine* gene (primer sequences *TaActine_*For: CACTGGAATGGTCAAGGCTG, *TaActine_*Rev: CTCCATGTC ATCCCAGTTG) was used as internal control and HI 1500 genotype (with minimum expression level) was used as a reference. For expression analysis, two biological replications for each genotype were performed and three technical replications were analyzed for each biological replication.

### Motif search in amplified promoter (regulatory) sequence

For motif search, promoter region involving ~1-kb segment upstream of the *TaGW2-6A* gene was examined using PLACE database (http://www.dna.affrc.go.jp/htdocs/PLACE/) [32].

### Development of functional marker

Phenotyping and genotypic data were used to identify the causal SNP (at -494bp). The causal SNP was then converted into a CAPS (cleaved amplified polymorphism sequence) marker. Restriction site was identified using dCAPS Finder 2.0 program. Promoter region of *TaGW2-6A* was first amplified using Hap-6A-P1_For and Hap-6A-P1_Rev, followed by a second PCR (primer pair: Hap-6A-P2_For and Hap-6A-P2_Rev [22]) to get smaller specific fragment. The amplified product (1μg DNA) of second PCR was then digested with *Fau*I (New England Biolabs) using 1 unit enzyme at 55^°^C for one h. The fragments resulting due to digestion were separated on 2% agarose gel.

## Results

### Variation for TGW and eight other agronomic traits in 207 Indian wheat genotypes

**Fig 1:**
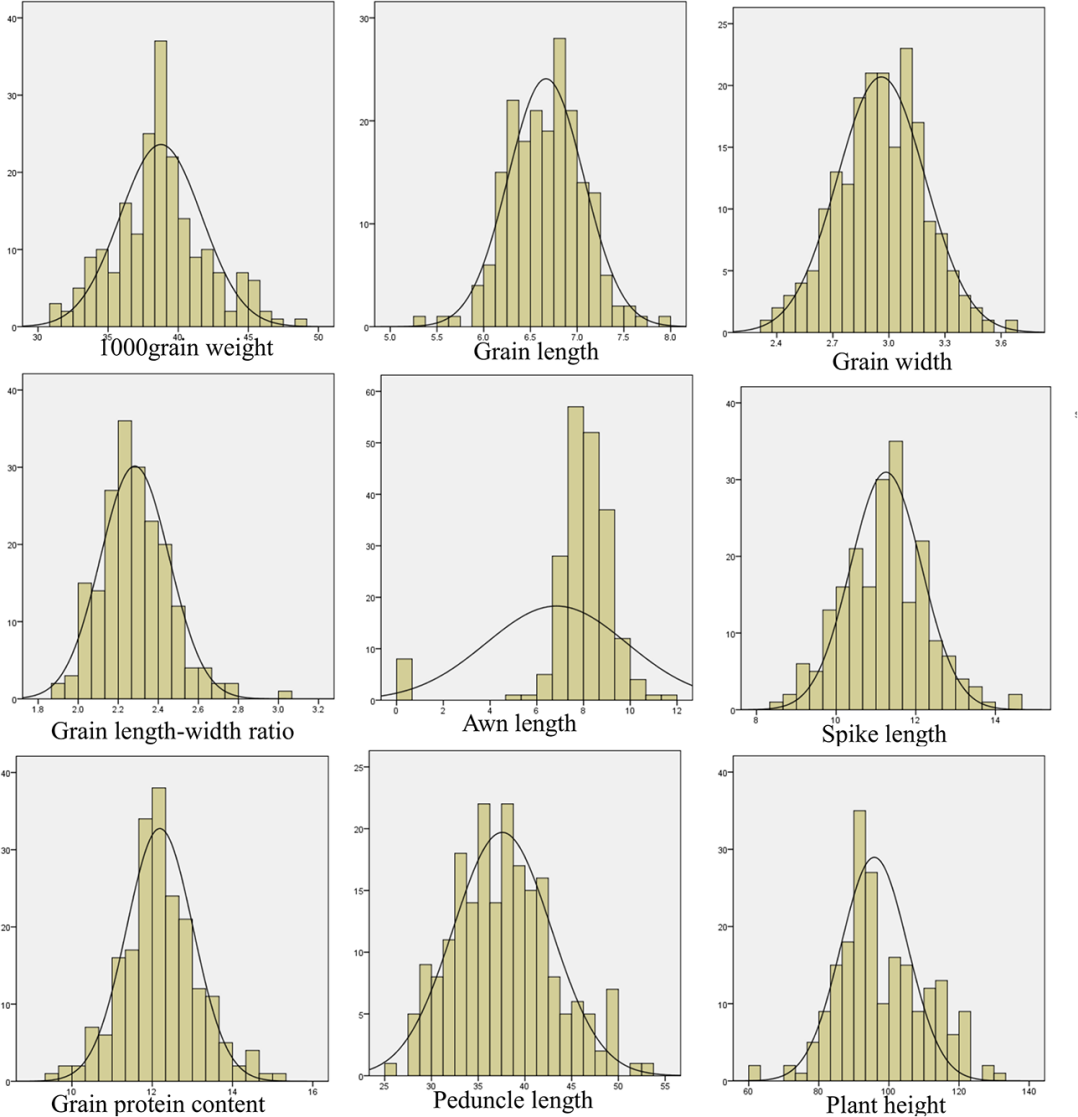
Frequency distribution curve for nine agronomic traits including 1000 grain weight in 207 Indian wheat genotypes used in association mapping study.

TGW in 207 Indian wheat genotypes ranged from 31.1 to 48.5 g with a mean of 38.7 g. The data gave a good fit to normal distribution with a standard deviation of 3.26 and coefficient of variation (CV) of 8.41%. Descriptive statistics for the remaining eight agronomic traits are presented in Table 1. Frequency distributions of genotypes with different class intervals of nine agronomic traits including TGW is presented in Fig 1.

**Table 1:** Descriptive statistics for 1000 grain weight and other agronomic traits.

Traits	Minimum	Maximum	Mean	Std. Deviation	CV (%)
1000 grain weight (g)	31.10	48.50	38.75	3.26	8.41
Grain length (mm)	5.33	7.97	6.67	0.40	6.05
Grain width (mm)	2.32	3.67	2.96	0.23	7.94
Length-width ratio	1.91	3.03	2.28	0.17	7.54
Awn length (cm)	0.00	11.59	7.83	1.82	23.19
Spike length (cm)	8.64	14.55	11.21	1.06	9.48
Grain protein content (%)	9.57	15.32	12.19	0.95	7.81
Peduncle length (cm)	25.93	53.10	37.69	5.35	14.20
Plant height (cm)	62.00	131.00	97.87	12.77	13.05

### Identification of two novel SNPs in the promoter region of *TaGW2-6A* in Indian wheat genotypes

**Fig 2:**
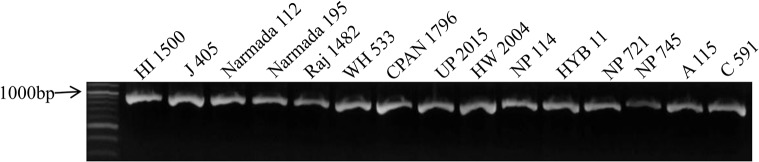
Representative gel picture of PCR amplification of promoter region of *TaGW2-6A* gene in 15 Indian wheat genotypes.

PCR amplification (Fig 2) and sequencing of the amplified promoter region of gene *TaGW2-6A* in 207 Indian wheat genotypes allowed identification of four SNPs, at positions -988bp, -739bp, -593bp and -494bp (S1 Fig) with minor allele frequencies of 7.2%, 15.0%, 14.0% and 6.3%, respectively. The details of the four SNPs are presented in Table 2. All the four SNPs were biallelic and all were transitions with G-A/A-G substitutions. Alignment of the amplified sequences of 207 Indian wheat genotypes and those reported by Su et al. [22] was done using multiple sequence alignment online software ClustalW2 (http://www.ebi.ac.uk/Tools/msa/clustalw2). The sequence alignment revealed that out of the four SNPs, two SNPs (at -988bp and at -494bp) were novel and were reported for the first time, while the remaining two SNPs at -739bp and -593bp positions were also reported earlier by Su et al. [22].

**Table 2:** Summary of four SNPs (identified by sequence alignment) with their position, variation, frequency and count.

Consensus position	Consensus	Allele variations	Frequencies	Counts
-988	G	G/A	92.8/7.2	192/15
-739	A	A/G	85.0/15.0	175/31
-593	G	G/A	86.0/14.0	178/29
-494	G	G/A	93.7/6.3	194/13

### Marker-trait association using individual SNP

Association mapping using general linear model (GLM) and Mann-Whitney U test revealed that out of the four SNPs identified during the present study, only one SNP (G/A at -494bp) was associated with TGW (Table 3); 13 genotypes with SNP allele having A at position 494 exhibited significantly higher TGW (41.1 g as against a mean TGW of 38.6 g in genotypes with SNP allele having G at position 494). None of the two SNPs earlier reported by Su et al. [22] showed association with TGW. SNP-494 was also found to be associated with grain length-width ratio and five other agronomic traits (awn length, spike length, grain protein content, peduncle length and plant height; Table 4), but not with grain length and grain width.

**Table 3:** Summary of marker-trait association using single SNP for 1000 grain weight. Significantly associated SNP is marked with *.

	General Linear Model			Mann-Whitney U test		Earlier report
Marker	F test	p-value	R^2^-value	U test	p-value	
SNP-988	0.248	0.619	0.001	1360	0.72	-
SNP-739	1.618	0.205	0.008	2221.5	0.1	[22]
SNP-593	1.017	0.314	0.005	2134.5	0.136	[22]
SNP-494*	7.621	0.006	0.036	594	0.001	-

**Table 4:** Marker-trait association of SNP-494 with 1000 grain weight and other eight agronomic traits.

Traits	General Linear Model				Mann-Whitney U test
	p-value	R^2^-value	G allele effect	A allele effect	p-value
1000 grain weight	0.006**	0.036	-2.54	0.00	0.001**
Grain length	0.306	-	-	-	0.328
Grain width	0.208	-	-	-	0.218
Length-width ratio	0.026*	0.026	0.12	0.00	0.016*
Awn length	0.048*	0.019	-1.03	0.00	0.003**
Spike length	0.005**	0.037	0.84	0.00	0.007**
Grain protein content (%)	0.036*	0.003	-0.59	0.00	0.021*
Peduncle length	0.000**	0.082	-6.31	0.00	0.000**
Plant height	0.000**	0.064	-13.31	0.00	0.000**

*^,^ ** significant at 0.05, and 0.01 levels, respectively.

### Haplotype analysis and their association with TGW

**Fig 3:**
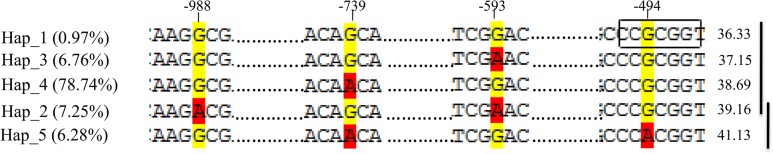
Five haplotypes with single nucleotide polymorphisms (SNPs) in the promoter regions of *TaGW2*-6A. Frequency of each haplotype is given in parentheses. SNPs are highlighted with yellow (G allele) and red (A allele) colours. CGCG motif is shown in a box. Significant difference of mean TGW (g) is represented by bars. Haplotypes covered by a single bar represent no significant difference for TGW and vice-versa.

Using four SNPs, following five haplotypes could be constituted [Fig 3: Hap1 (G_G_G_G), Hap2 (A_G_A_G), Hap3 (G_G_A_G), Hap4 (G_A_G_G_) and Hap5 (G_A_G_A)]. Out of 207 genotypes, Hap1 occured in 2 (0.97%) genotypes with a mean TGW of 36.33 g, Hap2 occurred in 15 (7.25%) genotypes with a mean TGW of 39.16 g, Hap3 occurred in 14 (6.76%) genotypes with a mean TGW of 38.7 g, Hap 4 occurred in 163 (78.74%) genotypes with a mean TGW of 38.7 g and Hap 5 occurred in 13 (6.28%) genotypes with a mean TGW of 41.13 g.

Analysis of variance (ANOVA) showed significant difference for TGW among 5 haplotypes (p < 0.01; Table 5), and also between Hap5 and the remaining four haplotypes (Hap5 vs others). A comparisons between pairs also showed that the mean TGW of Hap5 was significantly higher than the mean TGW of Hap1, Hap3 and Hap4; however no significant difference for TGW was observed between Hap5 and Hap2 (Fig 3).

**Table 5:** Analysis of variance for TGW using five different haplotypes.

Source	DF*	Mean Square	F Value	p-value	R^2^-value
Haplotype	4	31.094	3.04	0.0183	0.056806
Hap 5 vs. Haps 1, 2, 3, 4	1	92.842	9.08	0.0029	
Error	202	10.223			

*DF = Degree of freedom

### SNPs and motifs in the promoter region of *TaGW2-6A*

We also analysed if any of the SNPs detected in the ~1 Kbp promoter region of *TaGW2-6A* during the present study had association with any specific motif. The analysis led to the identification of several putative binding sites within the above region of promoter that was analysed during the present study (see S2 Fig). Out of the four SNPs, SNP -494 showing significant association with TGW was located in the ‘CGCG’ motif (see Fig 3).

### Relationship among SNP-494, *TaGW2-6A* expression and TGW

**Fig 4:**
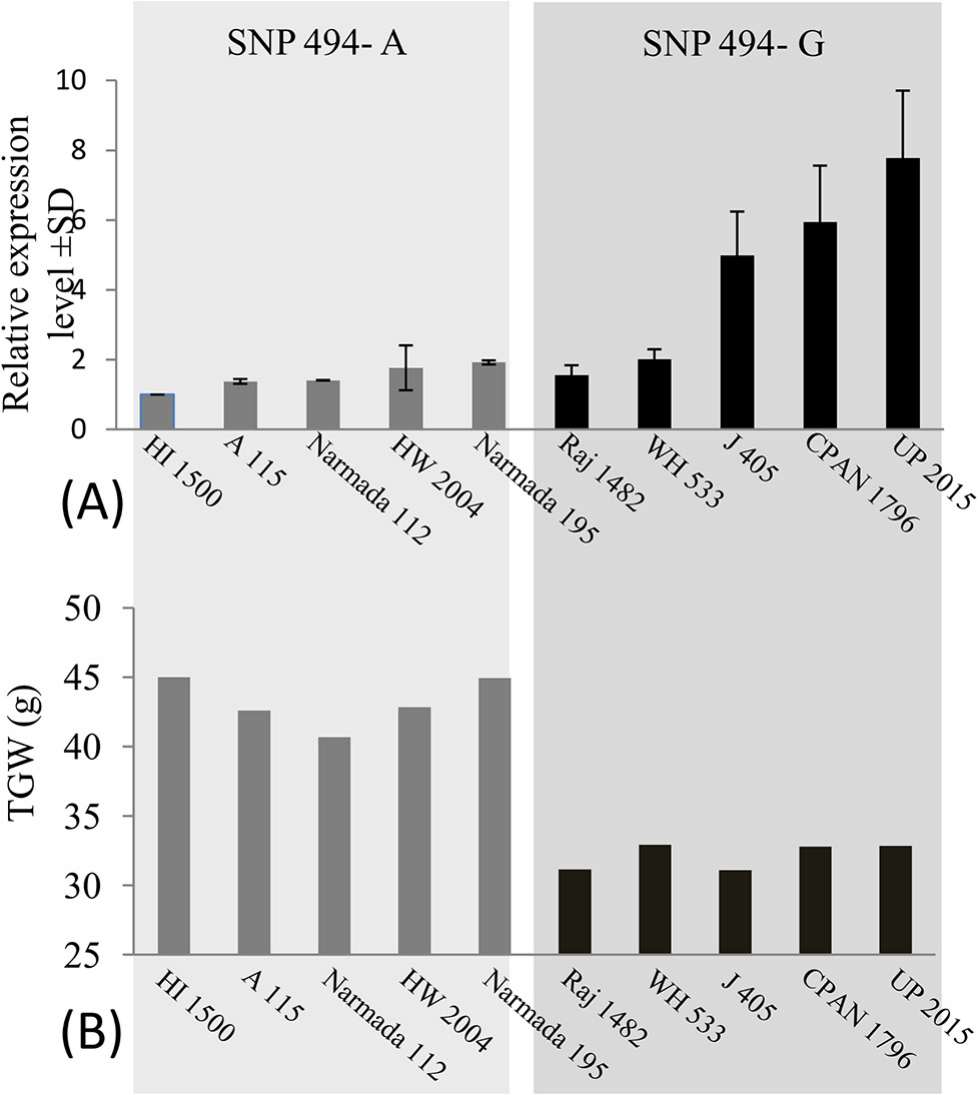
Bar diagrams showing (A) relative expression level of *TaGW2-6A* in immature seeds at 15dpf. Actin gene was used as the endogenous control and variety HI 1500 used as reference; (B) TGW of varieties having SNP-494_A on the left panel and those with SNP-494_G in the right panel.

**Fig 5:**
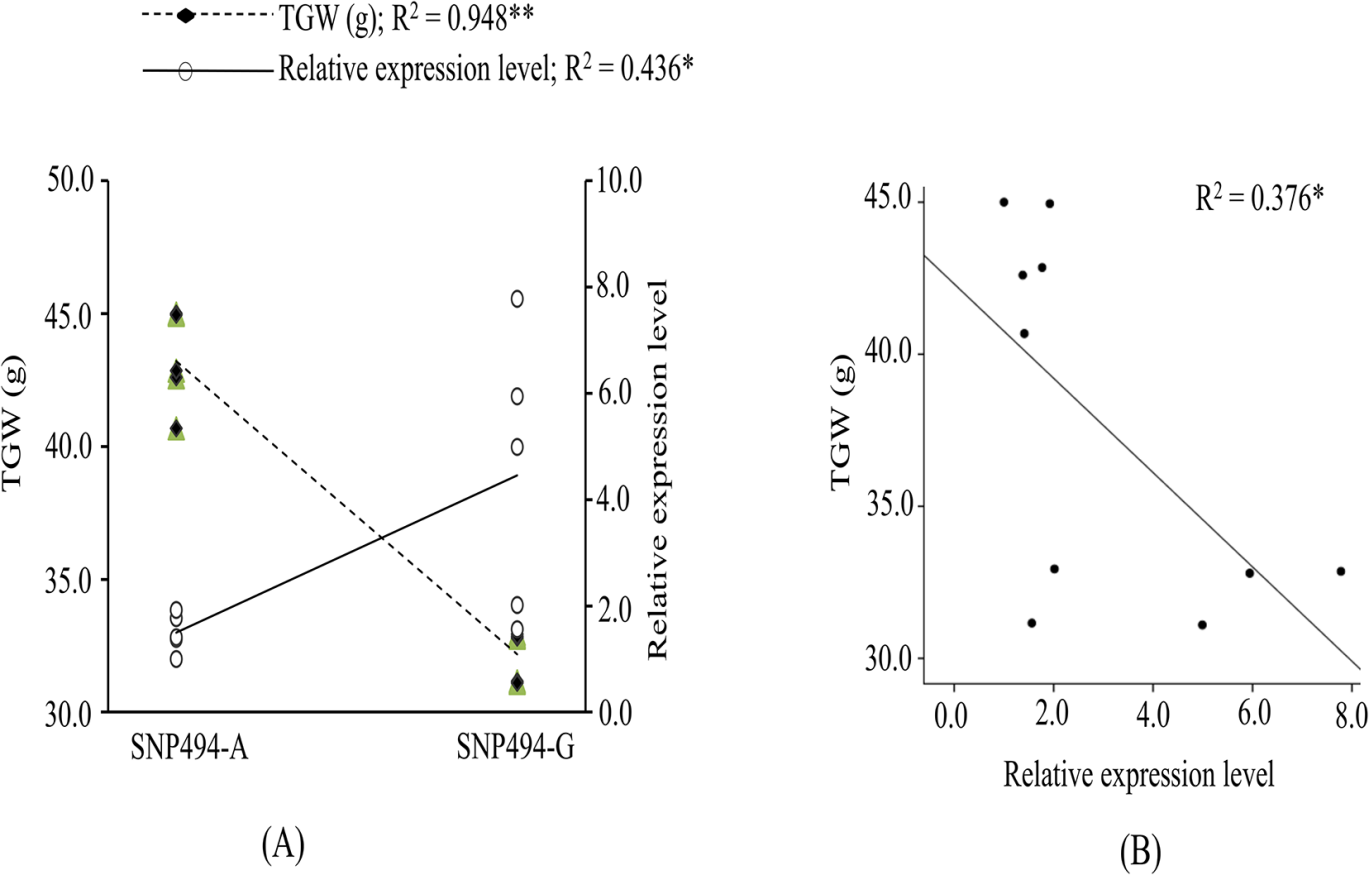
Plot showing significant regression of (A) *TaGW2-6A* expression level and thousand grain weight (TGW) in genotypes with two different alleles (A and G) of SNP-494 identified in promoter region, and (B) TGW with relative expression level in same genotypes. * and ** indicate significance at 0.05 and 0.01 levels, respectively.

Association of SNP-494 with expression of *TaGW2-6A* was also examined using five genotypes each with alleles A and G of this SNP. Genotypes with SNP-494_A had expression level, which was 1.0 to 1.9 fold (average = 1.49 fold), and the genotypes with SNP-494_G had expression level, which was 1.5 to 7.7 fold (average = 4.45 fold) relative to expression in HI 1500, used as a reference (see Fig 4). There was not much variation in the expression level among the five genotypes with SNP-494_A, although the expression level in the five genotypes with SNP-494_G differed markedly. Regression of the expression level of the gene *TaGW2-6A* and TGW on SNP-494 genotypes was significant, with A allele having significantly lower expression and higher TGW relative to that in genotypes with G allele (Fig 5A). TGW also exhibited a significant regression on the expression of the gene *TaGW2-6A*, suggesting that the expression of the gene *TaGW2-6A* has negative association with TGW (Fig 5B).

### Development of functional marker for utilization of *TaGW2-6A* for MAS

**Fig 6:**
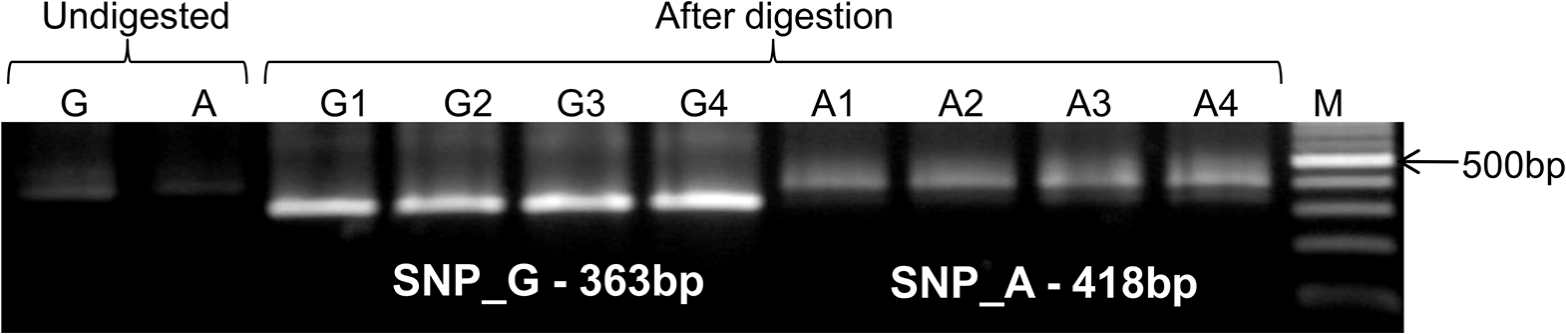
Validation of CAPS in genotypes with causal SNP_G and SNP_A on 2% agarose gel. G and A represent undigested PCR products of genotypes with SNP_G and SNP_A; G1–G4 and A1–A4 are genotypes with SNP_G and SNP_A after digestion with *FauI*, M = marker.

The causal SNP (SNP-494) was converted into a CAPS (cleaved amplified polymorphism sequence) marker to distinguish the alleles of *TaGW2-6A*. After digestion of the PCR product by *Fau*I, a length polymorphism (363-bp vs 418-bp) was observed in the cleavage products, which could be easily distinguished on agarose gels (Fig 6).

## Discussion

Two hundred seven (207) Indian wheat genotypes used in the present study were released over a period of ~100 (1910–2006) years and captured almost the entire genetic variation in TGW among Indian wheat genotypes. TGW in these 207 genotypes was normally distributed suggesting suitability of the mapping panel for conducting candidate gene-based association analysis. Similar candidate gene-based association mapping studies involving TGW and grain length were earlier conducted in China mainly using Chinese wheat germpalsm [33–35].

In the present study, we focused on sequence polymorphism in the promoter region of the gene *TaGW2-6A*, and detected two novel SNPs in this region. However, an insertion of a single base (T) was also earlier reported in the coding region, generating a pre-mature stop codon [23]. Thus, altogether, four SNPs in the promoter region and one insertion in the coding region of *TaGW2-6A*, are now known (present study and two earlier studies [22–23]). This supports the prevalent view that more SNPs occur in the promoter region than in the coding region of individual genes [22]. In future, more SNPs, indels and desirable haplotypes are likely to be identified, if screening of world wheat collection is undertaken. This variability in *TaGW2-6A* and similar other genes involved in grain weight may prove useful for the improvement of grain weight and related traits in bread wheat.

### SNPs and haplotype associated with grain weight

During the present study, association of TGW with only one novel SNP that occurred in the promoter region of *TaGW2-6A*, was detected. This SNP was available at -494bp position within the promoter; the other SNP that occurred at -593bp position was also reported earlier by Su et al. [22], but was not found to be associated with TGW during the present study. The association of SNP-494 suggested an involvement of this particular SNP in regulation of the expression of gene *TaGW2-6A*, as also indicated by the results of expression analysis conducted during the present study. Association of an insertion in the coding region of *TaGW2-6A* with grain weight was also reported in an earlier study [23], but could not be confirmed during the present study, which focused on the promoter region only.

Several earlier reports are available on candidate gene-based sssociation studies in wheat involving a variety of traits including TGW and grain length [33–35]; In an earlier study, association of two SNPs in the promoter region of the gene *TaGW2-6A* with TGW was reported in a Chinese wheat collection [22]. During the present study, similar information on this gene in Indian wheat germplasm was collected, which led to the identification of four SNPs including a novel SNP in the promoter region showing association with grain size including TGW and length-width ratio. Simultaneous association of this novel SNP with five other agronomic traits suggested that *TaGW2-6A* was also involved in controlling agronomic traits other than grain size. A user-friendly CAPS marker for the causal SNP was also developed for exploitation of the variation in *TaGW2-6A* gene for improvement in TGW and other associated agronomic traits through marker-assisted selection (MAS) in wheat. During the present study, only five of the 16 possible haplotypes (involving four SNPs) were available. A failure to detect all the possible haplotypes may be attributed to small population size as well as strong LD. Using the above five haplotypes, we conducted haplotype-based marker-trait association analysis to study intragenic interaction. Of all the five haplotypes, Hap5 (G_A_G_A) had significantly higher TGW than other haplotypes except Hap2, which did not show any significant difference from Hap5 (Fig 3). This suggested presence of some intragenic interaction among at least some of the SNPs.

### SNP in CGCG motif of promoter region and the putative pathway

**Fig 7:**
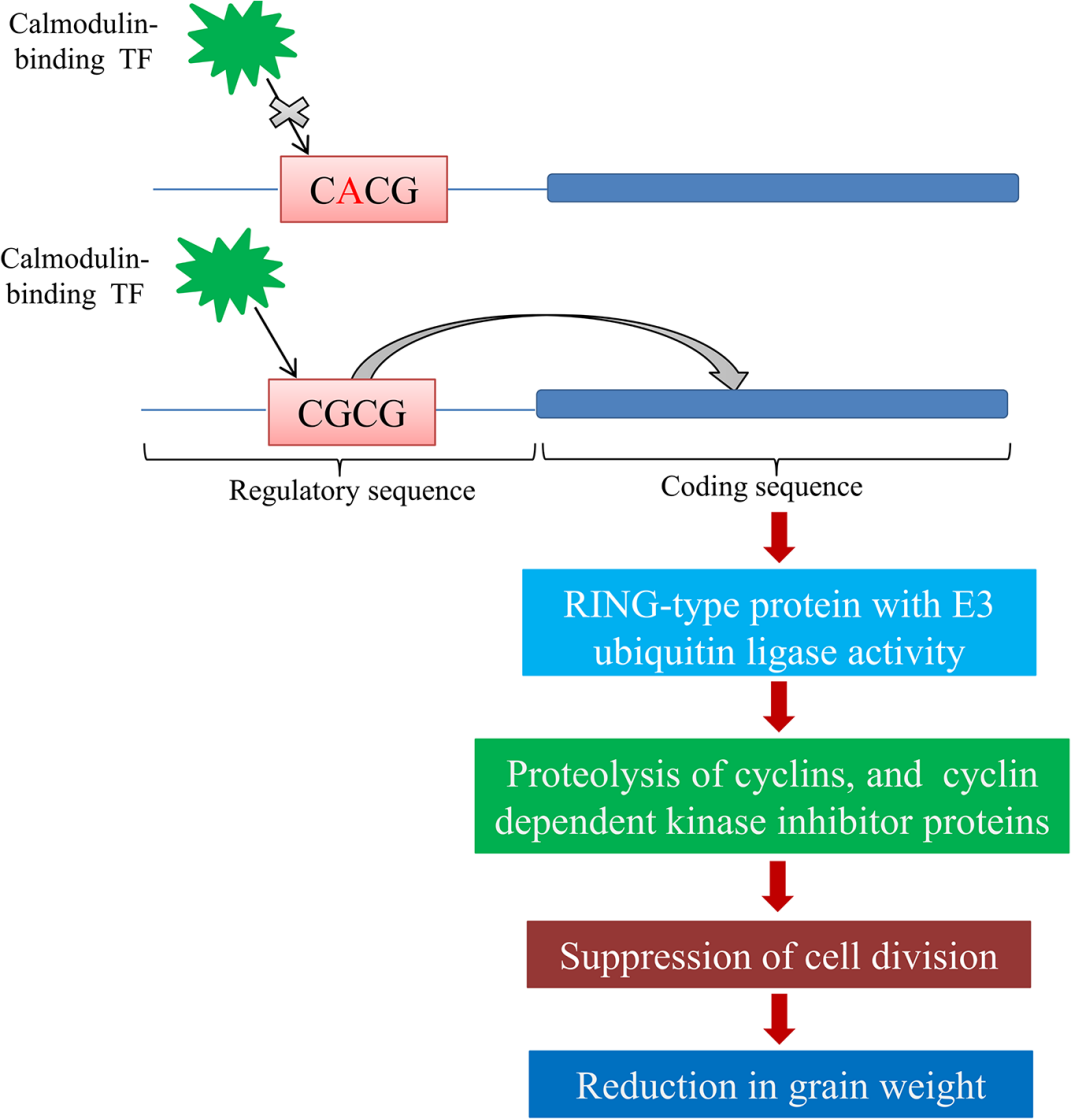
A hypothetical pathway of CGCG mediated regulation of *TaGW2-6A*.

The CGCG motif is an important motif, which occurs as a cis-regulatory element within the promoter of many genes that are under Ca^2+^/calmodulin (CAM) regulation [36–38], and provides a site for the binding of a calmodulin-binding transcription factor [36, 38]. We detected two “C**CGCG**G” motifs in the promoter region of *TaGW2-6A*, one at -810 bp and other at -495bp. The presence of more than one Ca^2+^/calmodulin-responsive cis regulatory elements in the promoter region of *TaGW2-6A* favours the possibility of a Ca^2+^-mediated regulation of *TaGW2*-6A gene expression in a manner similar to that of calcium-dependent protein kinases (CDPKs). In rice, a CDPK with calmodulin like domain (SPK) has been shown to be involved in accumulation of storage products during seed development [39]. It is possible that a similar mechanism is involved in the regulation of *TaGW2-6A* in wheat also. Interestingly, the SNP-494 was located in one of the two “C**G**CG” motifs (at -495bp). Expression analysis also revealed that SNP-494 was involved in the regulation of the expression of *TaGW2-6A*. In view of the above, a hypothetical pathway of CGCG mediated regulation of *TaGW2-6A* gene is proposed (Fig 7). The pathway indicate that a calmodulin-binding transcription factor binds to the cis regulatory CGCG motif allowing enhanced expression of *TaGW2-*6A, which encodes a ring type protein with E3 ubiquitin ligase activity. The ring type protein with E3 ubiquitin ligase activity bind with substrates like cyclins, and cyclin dependent kinase inhibitor proteins, allowing the proteolysis of these important proteins, which have a key role in the progression of the cell division cycle [40]. This leads to suppression of cell division and consequent reduction in TGW. Reverse is the case if CGCG box got mutated into CACG, which leads to reduced expression of *TaGW2-6A*, thus leading to higher grain weight.

Of the 207 wheat genotypes examined during the present study, as many as 194 genotypes carried CGCG motif with G allele and the remaining set of 13 genotypes had CACG motif with ‘A’ allele at the SNP-494 locus. This suggested that the motif C**G**CG (carrying SNP allele G) is the predominant wild type and the other motif C**A**CG carrying the allele **A** evolved during the course of evolution. A perusal of TGW values of these two sets of haplotypes revealed that haplotype with C**A**CG motif had significantly higher mean TGW than the haplotype with C**G**CG; this suggested that C**A**CG motif might have evolved later due to selection for higher grain weight.

### *TaGW2-6A* as a negative regulator of grain size

*TaGW2-6A* in wheat and its homologues in rice and maize are constitutively expressed [22, 41–42]. In rice, the gene *OsGW2* for grain size encodes a RING-type protein with E3 ubiquitin ligase that negatively regulates grain width through control of cell division in the spikelet hull. Loss-of-function mutations in the coding sequence, or interference with the expression level of *OsGW2*, resulted in enhanced grain width, grain weight and grain yield [41]. In wheat, two earlier studies involving *TaGW2-6A* concluded that like rice gene *OsGW2*, its wheat otrthologue *TaGW2-6A* is also a negative regulator of grain-width and grain-weight [22, 23]. The present study also suggested negative regulation of grain size in wheat by *TaGW2-6A*. However, Bednarek et al. [24] reported that RNAi-based down-regulation of *TaGW2* expression resulted in a significant reduction in final grain weight and size. Following may be the possible reasons for these apparently opposite results: (1) the gene *TaGW2-6A* may have different genetic backgrounds in the genotypes used in different studies; (2) there may be other genes, which may be silenced during the study conducted by Bednarek et al. [24], who used full-length sequence of ~1275bp to construct the RNAi cassette which might have resulted in off-target effects to silence other genes; (3) three homoeologues of *TaGW2* may have different effects on grain weight, so that silencing of all the three genes might result in reduction in grain weight: this contention received support from a recent study [30], where it has been reported that transcript abundance of *TaGW2-6A* is negatively associated with the grain width, but the transcript levels of *TaGW-2B* and *TaGW-2D* were positively associated with the grain width in the same bread wheat accessions. This suggested that triplicate homoeologues of *TaGW2* might have different functions in grain development, and that there is a balance among three genes finally determining the grain size in bread wheat.

### *TaGW2-6A* with other yield related genes in wheat

Beside *TaGW2*, three other genes, namely *TaGASR7-A1*, *TaGS-D1* and *6-SFT-A2* which control grain weight and/or length have been recently reported [33–35]. However, there must be a number of other yield-related important genes controlling grain weight in wheat. A number of such genes (e.g., *GS3*, *GW5*, *GW8*, *TGW6*, *Ghd7* and *GIF1*) have actually been isolated and cloned in rice [43–48] and there is no reason why orthologues of these genes may not be available in wheat. The availability of draft genome sequence in wheat should facilitate prediction and cloning of a number of these yield-related genes, so that it will be possible to identify favourable alleles and develop functional markers for these genes. This knowledge about yield related genes including *TaGW2* used for the present study may prove useful for development of high yielding wheat cultivars through marker-assisted selection.

## Conclusions

A novel SNP (SNP-494) was identified in the promoter region of the gene *TaGW2-6A*, which significantly affects TGW, grain length-width ratio and five other agronomic traits in wheat. This SNP was also a part of a haplotype and was located in an important motif (CGCG), which may possibly be a site for one or more calmodulin-binding transcription factors and eventually may be involved in regulation of the expression of the *TaGW2-6A* gene. This SNP was found to regulate the expression of the gene *TaGW2-6A*. The findings of the present study provide an initial step toward dissecting the molecular mechanism underlying seed development and TGW in wheat. The functional CAPS marker developed for causal SNP during the present study is recommended for use in marker-assisted selection for improvement of TGW along with other agronomic traits in wheat.

## Supporting Information

S1 Fig Five haplotypes with single nucleotide polymorphisms compositions in the promoter regions of *TaGW2*-6A. SNPs are highlighted with yellow (G allele) and red (A allele). SNP involving CGCG motif is represented with box.
(DOCX)

S2 Fig Motifs present in the promoter sequence of *TaGW2-6A*. (DOCX)
