# Supplementary material for: Identification of Novel SNP in Promoter Sequence of TaGW2-6A Associated with Grain Weight and Other Agronomic Traits in Wheat (Triticum aestivum L.)
Source: PLoS One. 2015 Jun 15;10(6):e0129400. doi: 10.1371/journal.pone.0129400 (PMC4468092; doi:10.1371/journal.pone.0129400)
Supplement: S1 Fig — SNPs are highlighted with yellow (G allele) and red (A allele). SNP involving CGCG motif is represented with box. (DOCX) [file pone.0129400.s001.docx]

Hap4_ TGCAATTTTCCGGAGATATAAATTCCTTATAGCCAATGTGGTGAACATAGCAAATTGATTCCCCCGGGTTTGATTCCATGTGCTCTAGCC -1134

Hap5_ TGCAATTTTCCGGAGATATAAATTCCTTATAGCCAATGTGGTGAACATAGCAAATTGATTCCCCCGGGTTTGATTCCATGTGCTCTAGCC -1134

Hap1_ TGCAATTTTCCGGAGATATAAATTCCTTATAGCCAATGTGGTGAACATAGCAAATTGATTCCCCCGGGTTTGATTCCATGTGCTCTAGCC -1134

Hap2_ TGCAATTTTCCGGAGATATAAATTCCTTATAGCCAATGTGGTGAACATAGCAAATTGATTCCCCCGGGTTTGATTCCATGTGCTCTAGCC -1134

Hap3_ TGCAATTTTCCGGAGATATAAATTCCTTATAGCCAATGTGGTGAACATAGCAAATTGATTCCCCCGGGTTTGATTCCATGTGCTCTAGCC -1134

Hap4_ AAAATAGATCAAATCAGCAAGATATCTTATGCTATGAATGGTGATAGTGGTCGCTCATGTTCATCTCGACTGCCGAAATCATGTGCCCTT -1044

Hap5_ AAAATAGATCAAATCAGCAAGATATCTTATGCTATGAATGGTGATAGTGGTCGCTCATGTTCATCTCGACTGCCGAAATCATGTGCCCTT -1044

Hap1_ AAAATAGATCAAATCAGCAAGATATCTTATGCTATGAATGGTGATAGTGGTCGCTCATGTTCATCTCGACTGCCGAAATCATGTGCCCTT -1044

Hap2_ AAAATAGATCAAATCAGCAAGATATCTTATGCTATGAATGGTGATAGTGGTCGCTCATGTTCATCTCGACTGCCGAAATCATGTGCCCTT -1044

Hap3_ AAAATAGATCAAATCAGCAAGATATCTTATGCTATGAATGGTGATAGTGGTCGCTCATGTTCATCTCGACTGCCGAAATCATGTGCCCTT -1044

Hap4_ AGCGGACGTTGTACTCCTCGGCAGTGGCCACTATCAACGAGCGGCGGCAGCCAAGGCGAAGGGATCCATGCAGATCGTGAGCAACCGTCC -954

Hap5_ AGCGGACGTTGTACTCCTCGGCAGTGGCCACTATCAACGAGCGGCGGCAGCCAAGGCGAAGGGATCCATGCAGATCGTGAGCAACCGTCC -954

Hap1_ AGCGGACGTTGTACTCCTCGGCAGTGGCCACTATCAACGAGCGGCGGCAGCCAAGGCGAAGGGATCCATGCAGATCGTGAGCAACCGTCC -954

Hap2_ AGCGGACGTTGTACTCCTCGGCAGTGGCCACTATCAACGAGCGGCGGCAGCCAAGACGAAGGGATCCATGCAGATCGTGAGCAACCGTCC -954

Hap3_ AGCGGACGTTGTACTCCTCGGCAGTGGCCACTATCAACGAGCGGCGGCAGCCAAGGCGAAGGGATCCATGCAGATCGTGAGCAACCGTCC -954

Hap4_ ATGCGTGGCTGCGGCAGACCGGAGCACGAGTAGGAGGCGGCAGATTCCACCATGGAAGGCCGAGGAGTGGCAAGGGTAGAGGTGGATGCA -864

Hap5_ ATGCGTGGCTGCGGCAGACCGGAGCACGAGTAGGAGGCGGCAGATTCCACCATGGAAGGCCGAGGAGTGGCAAGGGTAGAGGTGGATGCA -864

Hap1_ ATGCGTGGCTGCGGCAGACCGGAGCACGAGTAGGAGGCGGCAGATTCCACCATGGAAGGCCGAGGAGTGGCAAGGGTAGAGGTGGATGCA -864

Hap2_ ATGCGTGGCTGCGGCAGACCGGAGCACGAGTAGGAGGCGGCAGATTCCACCATGGAAGGCCGAGGAGTGGCAAGGGTAGAGGTGGATGCA -864

Hap3_ ATGCGTGGCTGCGGCAGACCGGAGCACGAGTAGGAGGCGGCAGATTCCACCATGGAAGGCCGAGGAGTGGCAAGGGTAGAGGTGGATGCA -864

Hap4_ GGAGGGAGGGGGGGGGGGAGAAAGGGCTGGTGCTATGGACCGCGGGAGGGGAGGACGTGCCAGTGACGAGGGAAGCGAAGGGCGGAGCGG -774

Hap5_ GGAGGGAGGGGGGGGGGGAGAAAGGGCTGGTGCTATGGACCGCGGGAGGGGAGGACGTGCCAGTGACGAGGGAAGCGAAGGGCGGAGCGG -774

Hap1_ GGAGGGAGGGGGGGGGGGAGAAAGGGCTGGTGCTATGGACCGCGGGAGGGGAGGACGTGCCAGTGACGAGGGAAGCGAAGGGCGGAGCGG -774

Hap2_ GGAGGGAGGGGGGGGGGGAGAAAGGGCTGGTGCTATGGACCGCGGGAGGGGAGGACGTGCCAGTGACGAGGGAAGCGAAGGGCGGAGCGG -774

Hap3_ GGAGGGAGGGGGGGGGGGAGAAAGGGCTGGTGCTATGGACCGCGGGAGGGGAGGACGTGCCAGTGACGAGGGAAGCGAAGGGCGGAGCGG -774

Su et al. 2011 GGTCGATGAGATCCCGTACAGCAGCTCGCAACAAACCCTAGCTCGCGCGAGAAGAGAGAGGGGATGTTCGGATCAA

Hap4_ CAGGAGGCCTGTCGGGTCGATGAGATCCCGTACAACAGCTCGCAACAAACCCTAGCTCGCGCGAGAAGAGAGAGGGGATGTTCGGATCAA -684

Hap5_ CAGGAGGCCTGTCGGGTCGATGAGATCCCGTACAACAGCTCGCAACAAACCCTAGCTCGCGCGAGAAGAGAGAGGGGATGTTCGGATCAA -684

Hap1_ CAGGAGGCCTGTCGGGTCGATGAGATCCCGTACAGCAGCTCGCAACAAACCCTAGCTCGCGCGAGAAGAGAGAGGGGATGTTCGGATCAA -684

Hap2_ CAGGAGGCCTGTCGGGTCGATGAGATCCCGTACAGCAGCTCGCAACAAACCCTAGCTCGCGCGAGAAGAGAGAGGGGATGTTCGGATCAA -684

Hap3_ CAGGAGGCCTGTCGGGTCGATGAGATCCCGTACAGCAGCTCGCAACAAACCCTAGCTCGCGCGAGAAGAGAGAGGGGATGTTCGGATCAA -684

AGAGAGGACGAGAGAAAACCGGCGTGGTAAGAAAAATCGATAAGGAAAGAACATCGTATGAGTGGAGAAGGGTGAGACGAAAATAAATCG

Hap4_ AGAGAGGACGAGAGAAAACCGGCGTGGTAAGAAAAATCGATAAGGAAAGAACATCGTATGAGTGGAGAAGGGTGAGACGAAAATAAATCG -594

Hap5_ AGAGAGGACGAGAGAAAACCGGCGTGGTAAGAAAAATCGATAAGGAAAGAACATCGTATGAGTGGAGAAGGGTGAGACGAAAATAAATCG -594

Hap1_ AGAGAGGACGAGAGAAAACCGGCGTGGTAAGAAAAATCGATAAGGAAAGAACATCGTATGAGTGGAGAAGGGTGAGACGAAAATAAATCG -594

Hap2_ AGAGAGGACGAGAGAAAACCGGCGTGGTAAGAAAAATCGATAAGGAAAGAACATCGTATGAGTGGAGAAGGGTGAGACGAAAATAAATCG -594

Hap3_ AGAGAGGACGAGAGAAAACCGGCGTGGTAAGAAAAATCGATAAGGAAAGAACATCGTATGAGTGGAGAAGGGTGAGACGAAAATAAATCG -594

GACGAAAATAATCATAAAGTGAAAGCTACCAAGTCCTTCTTTAAAAGTAGAGATCACATATTCGCTTAGAGGAA

Hap4_ GACGAAAATAATCATAAAGTGAAAGCTACCAAGTCCTTCTTTAAAAGTAGAGATCACATATTCGCTTAGAGGAAAGATGAAGGGGTAGGT -504

Hap5_ GACGAAAATAATCATAAAGTGAAAGCTACCAAGTCCTTCTTTAAAAGTAGAGATCACATATTCGCTTAGAGGAAAGATGAAGGGGTAGGT -504

Hap1_ GACGAAAATAATCATAAAGTGAAAGCTACCAAGTCCTTCTTTAAAAGTAGAGATCACATATTCGCTTAGAGGAAAGATGAAGGGGTAGGT -504

Hap2_ AACGAAAATAATCATAAAGTGAAAGCTACCAAGTCCTTCTTTAAAAGTAGAGATCACATATTCGCTTAGAGGAAAGATGAAGGGGTAGGT -504

Hap3_ AACGAAAATAATCATAAAGTGAAAGCTACCAAGTCCTTCTTTAAAAGTAGAGATCACATATTCGCTTAGAGGAAAGATGAAGGGGTAGGT -504

Hap4_ GATGCGCCCGCGGTGATGCACTCATCATGTCGCTTCCCATTTACGAAAGCATTACCTATGTTTATCAAGCGTTACATGGGATAGGGGATA -414

Hap5_ GATGCGCCCACGGTGATGCACTCATCATGTCGCTTCCCATTTACGAAAGCATTACCTATGTTTATCAAGCGTTACATGGGATAGGGGATA -414

Hap1_ GATGCGCCCGCGGTGATGCACTCATCATGTCGCTTCCCATTTACGAAAGCATTACCTATGTTTATCAAGCGTTACATGGGATAGGGGATA -414

Hap2_ GATGCGCCCGCGGTGATGCACTCATCATGTCGCTTCCCATTTACGAAAGCATTACCTATGTTTATCAAGCGTTACATGGGATAGGGGATA -414

Hap3_ GATGCGCCCGCGGTGATGCACTCATCATGTCGCTTCCCATTTACGAAAGCATTACCTATGTTTATCAAGCGTTACATGGGATAGGGGATA -414

Hap4_ TACACAAACATGTTCCTAAATTAATTAAAAAAAACATGTTTCCTAAATTGTGACACAGATCGATGGTCCAAGAATTTAAGCGGCCACACAA -323

Hap5_ TACACAAACATGTTCCTAAATTAATTAAAAAAAACATGTTTCCTAAATTGTGACACAGATCGATGGTCCAAGAATTTAAGCGGCCACACAA -323

Hap1_ TACACAAACATGTTCCTAAATTAATTAAAAAAAACATGTTTCCTAAATTGTGACACAGATCGATGGTCCAAGAATTTAAGCGGCCACACAA -323

Hap2_ TACACAAACATGTTCCTAAATTAATTAAAAAAAACATGTTTCCTAAATTGTGACACAGATCGATGGTCCAAGAATTTAAGCGGCCACACAA -323

Hap3_ TACACAAACATGTTCCTAAATTAATTAAAAAAAACATGTTTCCTAAATTGTGACACAGATCGATGGTCCAAGAATTTAAGCGGCCACACAA -323

S1 Fig. Five haplotypes with single nucleotide polymorphisms compositions in the promoter regions of *TaGW2*-6A. SNPs are highlighted with yellow (G allele) and red (A allele). SNP involving CGCG motif is represented with box.
