# Supplementary material for: Identification of Novel SNP in Promoter Sequence of TaGW2-6A Associated with Grain Weight and Other Agronomic Traits in Wheat (Triticum aestivum L.)
Source: PLoS One. 2015 Jun 15;10(6):e0129400. doi: 10.1371/journal.pone.0129400 (PMC4468092; doi:10.1371/journal.pone.0129400)
Supplement: S2 Fig — (DOCX) [file pone.0129400.s002.docx]

-1223 TGCAATTTTCCGGAGATATAAATTCCTTATAGCCAATGTGGTGAACATAG

(+)CAATBOX1 [S000028](http://www.dna.affrc.go.jp/sigscan/disp.cgi?S000028)

(-)GT1CONSENSUS [S000198](http://www.dna.affrc.go.jp/sigscan/disp.cgi?S000198)

(-)GT1CONSENSUS [S000198](http://www.dna.affrc.go.jp/sigscan/disp.cgi?S000198)

(+)GATABOX [S000039](http://www.dna.affrc.go.jp/sigscan/disp.cgi?S000039)

(+)TATABOX2 [S000109](http://www.dna.affrc.go.jp/sigscan/disp.cgi?S000109)

(+)CCAATBOX1 [S000030](http://www.dna.affrc.go.jp/sigscan/disp.cgi?S000030)

(+)LEAFYATAG [S000432](http://www.dna.affrc.go.jp/sigscan/disp.cgi?S000432)

(+)CAATBOX1 [S000028](http://www.dna.affrc.go.jp/sigscan/disp.cgi?S000028)

(+)GTGANTG10 [S000378](http://www.dna.affrc.go.jp/sigscan/disp.cgi?S000378)

-1173 CAAATTGATTCCCCCGGGTTTGATTCCATGTGCTCTAGCCAAAATAGATC

(-)CAATBOX1 [S000028](http://www.dna.affrc.go.jp/sigscan/disp.cgi?S000028)

(+)ARR1AT [S000454](http://www.dna.affrc.go.jp/sigscan/disp.cgi?S000454)

(+)E2FCONSENSUS [S000476](http://www.dna.affrc.go.jp/sigscan/disp.cgi?S000476)

(+)ARR1AT [S000454](http://www.dna.affrc.go.jp/sigscan/disp.cgi?S000454)

(+)EBOXBNNAPA [S000144](http://www.dna.affrc.go.jp/sigscan/disp.cgi?S000144)

(+)MYCATERD1 [S000413](http://www.dna.affrc.go.jp/sigscan/disp.cgi?S000413)

(+)MYCCONSENSUSAT [S000407](http://www.dna.affrc.go.jp/sigscan/disp.cgi?S000407)

(-)EBOXBNNAPA [S000144](http://www.dna.affrc.go.jp/sigscan/disp.cgi?S000144)

(-)MYCATRD22 [S000174](http://www.dna.affrc.go.jp/sigscan/disp.cgi?S000174)

(-)MYCCONSENSUSAT [S000407](http://www.dna.affrc.go.jp/sigscan/disp.cgi?S000407)

-1123 AAATCAGCAAGATATCTTATGCTATGAATGGTGATAGTGGTCGCTCATGT

(-)ARR1AT [S000454](http://www.dna.affrc.go.jp/sigscan/disp.cgi?S000454)

(+)GATABOX [S000039](http://www.dna.affrc.go.jp/sigscan/disp.cgi?S000039)

(-)GATABOX [S000039](http://www.dna.affrc.go.jp/sigscan/disp.cgi?S000039)

(+)GTGANTG10 [S000378](http://www.dna.affrc.go.jp/sigscan/disp.cgi?S000378)

(+)GATABOX [S000039](http://www.dna.affrc.go.jp/sigscan/disp.cgi?S000039)

(-)CACTFTPPCA1 [S000449](http://www.dna.affrc.go.jp/sigscan/disp.cgi?S000449)

-1073 TCATCTCGACTGCCGAAATCATGTGCCCTTAGCGGACGTTGTACTCCTCG

(+)LTRE1HVBLT49 [S000250](http://www.dna.affrc.go.jp/sigscan/disp.cgi?S000250)

(-)ARR1AT [S000454](http://www.dna.affrc.go.jp/sigscan/disp.cgi?S000454)

(+)EBOXBNNAPA [S000144](http://www.dna.affrc.go.jp/sigscan/disp.cgi?S000144)

(+)MYCATERD1 [S000413](http://www.dna.affrc.go.jp/sigscan/disp.cgi?S000413)

(+)MYCCONSENSUSAT [S000407](http://www.dna.affrc.go.jp/sigscan/disp.cgi?S000407)

(-)EBOXBNNAPA [S000144](http://www.dna.affrc.go.jp/sigscan/disp.cgi?S000144)

(-)MYCATRD22 [S000174](http://www.dna.affrc.go.jp/sigscan/disp.cgi?S000174)

(-)MYCCONSENSUSAT [S000407](http://www.dna.affrc.go.jp/sigscan/disp.cgi?S000407)

(+)MNF1ZMPPC1 [S000251](http://www.dna.affrc.go.jp/sigscan/disp.cgi?S000251)

(-)ABREOSRAB21 [S000012](http://www.dna.affrc.go.jp/sigscan/disp.cgi?S000012)

(+)ACGTATERD1 [S000415](http://www.dna.affrc.go.jp/sigscan/disp.cgi?S000415)

(-)ACGTATERD1 [S000415](http://www.dna.affrc.go.jp/sigscan/disp.cgi?S000415)

(+)CURECORECR [S000493](http://www.dna.affrc.go.jp/sigscan/disp.cgi?S000493)

(-)CURECORECR [S000493](http://www.dna.affrc.go.jp/sigscan/disp.cgi?S000493)

(+)CACTFTPPCA1 [S000449](http://www.dna.affrc.go.jp/sigscan/disp.cgi?S000449)

-1023 GCAGTGGCCACTATCAACGAGCGGCGGCAGCCAAGGCGAAGGGATCCATG

(-)CACTFTPPCA1 [S000449](http://www.dna.affrc.go.jp/sigscan/disp.cgi?S000449)

(-)SORLIP1AT [S000482](http://www.dna.affrc.go.jp/sigscan/disp.cgi?S000482)

(+)SORLIP1AT [S000482](http://www.dna.affrc.go.jp/sigscan/disp.cgi?S000482)

(+)CACTFTPPCA1 [S000449](http://www.dna.affrc.go.jp/sigscan/disp.cgi?S000449)

(-)GATABOX [S000039](http://www.dna.affrc.go.jp/sigscan/disp.cgi?S000039)

(-)GCCCORE [S000430](http://www.dna.affrc.go.jp/sigscan/disp.cgi?S000430)

(+)RYREPEATBNNAPA [S000264](http://www.dna.affrc.go.jp/sigscan/disp.cgi?S000264)

-973 CAGATCGTGAGCAACCGTCCATGCGTGGCTGCGGCAGACCGGAGCACGAG

(-)RHERPATEXPA7 [S000512](http://www.dna.affrc.go.jp/sigscan/disp.cgi?S000512)

(+)GTGANTG10 [S000378](http://www.dna.affrc.go.jp/sigscan/disp.cgi?S000378)

(-)MYBCORE [S000176](http://www.dna.affrc.go.jp/sigscan/disp.cgi?S000176)

(+)PALBOXAPC [S000137](http://www.dna.affrc.go.jp/sigscan/disp.cgi?S000137)

(-)CMSRE1IBSPOA [S000511](http://www.dna.affrc.go.jp/sigscan/disp.cgi?S000511)

(-)UPRMOTIFIIAT [S000426](http://www.dna.affrc.go.jp/sigscan/disp.cgi?S000426)

(-)SORLIP1AT [S000482](http://www.dna.affrc.go.jp/sigscan/disp.cgi?S000482)

(+)RHERPATEXPA7 [S000512](http://www.dna.affrc.go.jp/sigscan/disp.cgi?S000512)

(-)CACTFTPPCA1 [S000449](http://www.dna.affrc.go.jp/sigscan/disp.cgi?S000449)

-923 TAGGAGGCGGCAGATTCCACCATGGAAGGCCGAGGAGTGGCAAGGGTAGA

(-)GCCCORE [S000430](http://www.dna.affrc.go.jp/sigscan/disp.cgi?S000430)

(+)ARR1AT [S000454](http://www.dna.affrc.go.jp/sigscan/disp.cgi?S000454)

(-)CACTFTPPCA1 [S000449](http://www.dna.affrc.go.jp/sigscan/disp.cgi?S000449)

(-)SORLIP1AT [S000482](http://www.dna.affrc.go.jp/sigscan/disp.cgi?S000482)

-873 GGTGGATGCAGGAGGGAGGGGGGGGGGGAGAAAGGGCTGGTGCTATGGAC

(+)INTRONLOWER [S000086](http://www.dna.affrc.go.jp/sigscan/disp.cgi?S000086)

(+)POLLEN1LELAT52 [S000245](http://www.dna.affrc.go.jp/sigscan/disp.cgi?S000245)

(+)DOFCOREZM [S000265](http://www.dna.affrc.go.jp/sigscan/disp.cgi?S000265)

(+)CGCGBOXAT [S000501](http://www.dna.affrc.go.jp/sigscan/disp.cgi?S000501)

(-)CGCGBOXAT [S000501](http://www.dna.affrc.go.jp/sigscan/disp.cgi?S000501)

-823 CGCGGGAGGGGAGGACGTGCCAGTGACGAGGGAAGCGAAGGGCGGAGCGG

(+)ABRELATERD1 [S000414](http://www.dna.affrc.go.jp/sigscan/disp.cgi?S000414)

(+)ACGTATERD1 [S000415](http://www.dna.affrc.go.jp/sigscan/disp.cgi?S000415)

(-)ACGTATERD1 [S000415](http://www.dna.affrc.go.jp/sigscan/disp.cgi?S000415)

(-)RHERPATEXPA7 [S000512](http://www.dna.affrc.go.jp/sigscan/disp.cgi?S000512)

(-)CACTFTPPCA1 [S000449](http://www.dna.affrc.go.jp/sigscan/disp.cgi?S000449)

(+)GTGANTG10 [S000378](http://www.dna.affrc.go.jp/sigscan/disp.cgi?S000378)

(+)ASF1MOTIFCAMV [S000024](http://www.dna.affrc.go.jp/sigscan/disp.cgi?S000024)

(+)WRKY71OS [S000447](http://www.dna.affrc.go.jp/sigscan/disp.cgi?S000447)

-773 CAGGAGGCCTGTCGGGTCGATGAGATCCCGTACAACAGCTCGCAACAAAC

(-)LTRECOREATCOR15 [S000153](http://www.dna.affrc.go.jp/sigscan/disp.cgi?S000153)

(-)CBFHV [S000497](http://www.dna.affrc.go.jp/sigscan/disp.cgi?S000497)

(+)CURECORECR [S000493](http://www.dna.affrc.go.jp/sigscan/disp.cgi?S000493)

(-)CURECORECR [S000493](http://www.dna.affrc.go.jp/sigscan/disp.cgi?S000493)

(+)RAV1AAT [S000314](http://www.dna.affrc.go.jp/sigscan/disp.cgi?S000314)

(-)MYBCORE [S000176](http://www.dna.affrc.go.jp/sigscan/disp.cgi?S000176)

(+)RAV1AAT [S000314](http://www.dna.affrc.go.jp/sigscan/disp.cgi?S000314)

(+)AACACOREOSGLUB1 [S000353](http://www.dna.affrc.go.jp/sigscan/disp.cgi?S000353)

(+)UP2ATMSD [S000472](http://www.dna.affrc.go.jp/sigscan/disp.cgi?S000472)

-723 CCTAGCTCGCGCGAGAAGAGAGAGGGGATGTTCGGATCAAAGAGAGGACG

(-)NODCON2GM [S000462](http://www.dna.affrc.go.jp/sigscan/disp.cgi?S000462)

(-)OSE2ROOTNODULE [S000468](http://www.dna.affrc.go.jp/sigscan/disp.cgi?S000468)

(+)DOFCOREZM [S000265](http://www.dna.affrc.go.jp/sigscan/disp.cgi?S000265)

(-)NODCON2GM [S000462](http://www.dna.affrc.go.jp/sigscan/disp.cgi?S000462)

(-)OSE2ROOTNODULE [S000468](http://www.dna.affrc.go.jp/sigscan/disp.cgi?S000468)

-673 AGAGAAAACCGGCGTGGTAAGAAAAATCGATAAGGAAAGAACATCGTATG

(+)POLLEN1LELAT52 [S000245](http://www.dna.affrc.go.jp/sigscan/disp.cgi?S000245)

(+)SV40COREENHAN [S000123](http://www.dna.affrc.go.jp/sigscan/disp.cgi?S000123)

(+)POLLEN1LELAT52 [S000245](http://www.dna.affrc.go.jp/sigscan/disp.cgi?S000245)

(+)GT1CONSENSUS [S000198](http://www.dna.affrc.go.jp/sigscan/disp.cgi?S000198)

(+)GT1GMSCAM4 [S000453](http://www.dna.affrc.go.jp/sigscan/disp.cgi?S000453)

(-)ARR1AT [S000454](http://www.dna.affrc.go.jp/sigscan/disp.cgi?S000454)

(+)GATABOX [S000039](http://www.dna.affrc.go.jp/sigscan/disp.cgi?S000039)

(+)IBOX [S000124](http://www.dna.affrc.go.jp/sigscan/disp.cgi?S000124)

(+)IBOXCORE [S000199](http://www.dna.affrc.go.jp/sigscan/disp.cgi?S000199)

(+)IBOXCORENT [S000424](http://www.dna.affrc.go.jp/sigscan/disp.cgi?S000424)

(+)DOFCOREZM [S000265](http://www.dna.affrc.go.jp/sigscan/disp.cgi?S000265)

(-)PREATPRODH [S000450](http://www.dna.affrc.go.jp/sigscan/disp.cgi?S000450)

-623 AGTGGAGAAGGGTGAGACGAAAATAAATCGGACGAAAATAATCATAAAGT

(-)CACTFTPPCA1 [S000449](http://www.dna.affrc.go.jp/sigscan/disp.cgi?S000449)

(+)GTGANTG10 [S000378](http://www.dna.affrc.go.jp/sigscan/disp.cgi?S000378)

(+)SURECOREATSULTR11 [S000499](http://www.dna.affrc.go.jp/sigscan/disp.cgi?S000499)

(+)GT1CONSENSUS [S000198](http://www.dna.affrc.go.jp/sigscan/disp.cgi?S000198)

(-)TATABOX5 [S000203](http://www.dna.affrc.go.jp/sigscan/disp.cgi?S000203)

(+)POLASIG1 [S000080](http://www.dna.affrc.go.jp/sigscan/disp.cgi?S000080)

(-)ARR1AT [S000454](http://www.dna.affrc.go.jp/sigscan/disp.cgi?S000454)

(+)GT1CONSENSUS [S000198](http://www.dna.affrc.go.jp/sigscan/disp.cgi?S000198)

(-)TATABOX5 [S000203](http://www.dna.affrc.go.jp/sigscan/disp.cgi?S000203)

(+)POLASIG3 [S000088](http://www.dna.affrc.go.jp/sigscan/disp.cgi?S000088)

(-)ARR1AT [S000454](http://www.dna.affrc.go.jp/sigscan/disp.cgi?S000454)

(+)TAAAGSTKST1 [S000387](http://www.dna.affrc.go.jp/sigscan/disp.cgi?S000387)

(-)NTBBF1ARROLB [S000273](http://www.dna.affrc.go.jp/sigscan/disp.cgi?S000273)

(+)DOFCOREZM [S000265](http://www.dna.affrc.go.jp/sigscan/disp.cgi?S000265)

(-)INRNTPSADB [S000395](http://www.dna.affrc.go.jp/sigscan/disp.cgi?S000395)

(-)CACTFTPPCA1 [S000449](http://www.dna.affrc.go.jp/sigscan/disp.cgi?S000449)

(+)GTGANTG10 [S000378](http://www.dna.affrc.go.jp/sigscan/disp.cgi?S000378)

-573 GAAAGCTACCAAGTCCTTCTTTAAAAGTAGAGATCACATATTCGCTTAGA

(+)DOFCOREZM [S000265](http://www.dna.affrc.go.jp/sigscan/disp.cgi?S000265)

(-)DOFCOREZM [S000265](http://www.dna.affrc.go.jp/sigscan/disp.cgi?S000265)

(-)TAAAGSTKST1 [S000387](http://www.dna.affrc.go.jp/sigscan/disp.cgi?S000387)

(+)DOFCOREZM [S000265](http://www.dna.affrc.go.jp/sigscan/disp.cgi?S000265)

(-)CACTFTPPCA1 [S000449](http://www.dna.affrc.go.jp/sigscan/disp.cgi?S000449)

(-)GTGANTG10 [S000378](http://www.dna.affrc.go.jp/sigscan/disp.cgi?S000378)

(+)ROOTMOTIFTAPOX1 [S000098](http://www.dna.affrc.go.jp/sigscan/disp.cgi?S000098)

-523 GGAAAGATGAAGGGGTAGGTGATGCGCCCGCGGTGATGCACTCATCATGT

(+)DOFCOREZM [S000265](http://www.dna.affrc.go.jp/sigscan/disp.cgi?S000265)

(+)NODCON1GM [S000461](http://www.dna.affrc.go.jp/sigscan/disp.cgi?S000461)

(+)OSE1ROOTNODULE [S000467](http://www.dna.affrc.go.jp/sigscan/disp.cgi?S000467)

(-)HBOXCONSENSUSPVCHS [S000200](http://www.dna.affrc.go.jp/sigscan/disp.cgi?S000200)

(-)BOXLCOREDCPAL [S000492](http://www.dna.affrc.go.jp/sigscan/disp.cgi?S000492)

(-)MYBPLANT [S000167](http://www.dna.affrc.go.jp/sigscan/disp.cgi?S000167)

(-)MYBPZM [S000179](http://www.dna.affrc.go.jp/sigscan/disp.cgi?S000179)

(+)GTGANTG10 [S000378](http://www.dna.affrc.go.jp/sigscan/disp.cgi?S000378)

(+)CGCGBOXAT [S000501](http://www.dna.affrc.go.jp/sigscan/disp.cgi?S000501)

(-)CGCGBOXAT [S000501](http://www.dna.affrc.go.jp/sigscan/disp.cgi?S000501)

(+)GTGANTG10 [S000378](http://www.dna.affrc.go.jp/sigscan/disp.cgi?S000378)

(+)CACTFTPPCA1 [S000449](http://www.dna.affrc.go.jp/sigscan/disp.cgi?S000449)

(+)PREATPRODH [S000450](http://www.dna.affrc.go.jp/sigscan/disp.cgi?S000450)

-473 CGCTTCCCATTTACGAAAGCATTACCTATGTTTATCAAGCGTTACATGGG

(+)DOFCOREZM [S000265](http://www.dna.affrc.go.jp/sigscan/disp.cgi?S000265)

(-)GT1CONSENSUS [S000198](http://www.dna.affrc.go.jp/sigscan/disp.cgi?S000198)

(-)GT1CONSENSUS [S000198](http://www.dna.affrc.go.jp/sigscan/disp.cgi?S000198)

(-)IBOXCORE [S000199](http://www.dna.affrc.go.jp/sigscan/disp.cgi?S000199)

(-)GATABOX [S000039](http://www.dna.affrc.go.jp/sigscan/disp.cgi?S000039)

(+)MYBST1 [S000180](http://www.dna.affrc.go.jp/sigscan/disp.cgi?S000180)

(+)GATABOX [S000039](http://www.dna.affrc.go.jp/sigscan/disp.cgi?S000039)

-423 ATAGGGGATATACACAAACATGTTCCTAAATTAATTAAAAAAAACATGTT

(+)MYBST1 [S000180](http://www.dna.affrc.go.jp/sigscan/disp.cgi?S000180)

(+)P1BS [S000459](http://www.dna.affrc.go.jp/sigscan/disp.cgi?S000459)

(-)P1BS [S000459](http://www.dna.affrc.go.jp/sigscan/disp.cgi?S000459)

(+)GATABOX [S000039](http://www.dna.affrc.go.jp/sigscan/disp.cgi?S000039)

(+)POLASIG2 [S000081](http://www.dna.affrc.go.jp/sigscan/disp.cgi?S000081)

-373 TCCTAAATTGTGACACAGATCGATGGTCCAAGAATTTAAGCGGCCACACA

(-)CAATBOX1 [S000028](http://www.dna.affrc.go.jp/sigscan/disp.cgi?S000028)

(-)TGTCACACMCUCUMISIN [S000422](http://www.dna.affrc.go.jp/sigscan/disp.cgi?S000422)

(+)GTGANTG10 [S000378](http://www.dna.affrc.go.jp/sigscan/disp.cgi?S000378)

(+)WRKY71OS [S000447](http://www.dna.affrc.go.jp/sigscan/disp.cgi?S000447)

(-)BIHD1OS [S000498](http://www.dna.affrc.go.jp/sigscan/disp.cgi?S000498)

(+)SORLIP1AT [S000482](http://www.dna.affrc.go.jp/sigscan/disp.cgi?S000482)

-323 A

S2 Fig. Motifs present in the promoter sequence of *TaGW2-6A.*
